# Prophylactic inhibition of neutrophil elastase prevents the development of chronic neuropathic pain in osteoarthritic mice

**DOI:** 10.1186/s12974-017-0944-0

**Published:** 2017-08-23

**Authors:** Milind M. Muley, Eugene Krustev, Allison R. Reid, Jason J. McDougall

**Affiliations:** Departments of Pharmacology and Anaesthesia, Pain Management & Perioperative Medicine, Dalhousie University, 5850 College Street, PO Box 15000, Halifax, Nova Scotia B3H 4R2 Canada

**Keywords:** Neutrophil elastase, Proteinase-activated receptor-2, Inflammation, Osteoarthritis, Pain, Peripheral neuropathy

## Abstract

**Background:**

A subset of osteoarthritis (OA) patients experience joint pain with neuropathic characteristics. Mediators such as neutrophil elastase, a serine proteinase, may be released during acute OA inflammatory flares. We have previously shown that local administration of neutrophil elastase causes joint inflammation and pain via activation of proteinase-activated receptor-2 (PAR2). The aim of this study was to examine the contribution of endogenous neutrophil elastase and PAR2 to the development of joint inflammation, pain, and neuropathy associated with monoiodoacetate (MIA)-induced experimental OA.

**Methods:**

MIA (0.3 mg/10 μl) was injected into the right knee joint of male C57BL/6 mice (20–34 g). Joint inflammation (edema, leukocyte kinetics), neutrophil elastase proteolytic activity, tactile allodynia, and saphenous nerve demyelination were assessed over 14 days post-injection. The effects of inhibiting neutrophil elastase during the early inflammatory phase of MIA (days 0 to 3) were determined using sivelestat (50 mg/kg i.p.) and serpinA1 (10 μg i.p.). Involvement of PAR2 in the development of MIA-induced joint inflammation and pain was studied using the PAR2 antagonist GB83 (5 μg i.p. days 0 to 1) and PAR2 knockout animals.

**Results:**

MIA caused an increase in neutrophil elastase proteolytic activity on day 1 (*P* < 0.0001), but not on day 14. MIA also generated a transient inflammatory response which peaked on day 1 (*P* < 0.01) then subsided over the 2-week time course. Joint pain appeared on day 1 and persisted to day 14 (*P* < 0.0001). By day 14, the saphenous nerve showed signs of demyelination. Early treatment with sivelestat and serpinA1 blocked the proteolytic activity of neutrophil elastase on day 1 (*P* < 0.001), and caused lasting improvements in joint inflammation, pain, and saphenous nerve damage (*P* < 0.05). MIA-induced synovitis was reversed by early treatment with GB83 and attenuated in PAR2 knockout mice (*P* < 0.05). PAR2 knockout mice also showed reduced MIA-induced joint pain (*P* < 0.0001) and less nerve demyelination (*P* = 0.81 compared to saline control).

**Conclusions:**

Neutrophil elastase and PAR2 contribute significantly to the development of joint inflammation, pain, and peripheral neuropathy associated with experimental OA, suggesting their potential as therapeutic targets.

## Background

While over 100 different types of joint-related conditions exist, osteoarthritis (OA) is the most prevalent [1]. In light of an ever aging population, the prevalence of OA will continue to increase rapidly in the coming years [2, 3]. OA is a degenerative disease that, traditionally, has been considered non-inflammatory; however, a growing body of evidence supports the presence of synovitis and the significant contribution it makes to the development of OA symptoms [4, 5]. Following injury, a multitude of inflammatory mediators are released into the joint that play a crucial role in the development of joint degeneration and pain. Various key cytokines like tumor necrosis factor alpha (TNF-α), interleukin 1 beta (IL-1β), and interleukin 6 (IL-6) are important for driving inflammation within the joint [6–8]. Interestingly, these inflammatory molecules can initiate neuronal damage and induce neuropathic pain responses [9, 10]. The peripheral nervous system also contributes to the initiation of joint inflammation during the early development of OA [11]. Various inflammatory neuropeptides such as substance P (SP) and calcitonin gene-related peptide (CGRP) are involved in this acute inflammatory OA response [11, 12], and these mediators are known to cause peripheral neuropathy [13–15]. Immune cells present at the site of inflammation release serine proteinases such as neutrophil elastase, cathepsin G, proteinase-3, and trypsin [16] that cause erosive destruction of joint cartilage and remodel subchondral bone [17]. Upon reaching a site of inflammation, neutrophils release neutrophil elastase which initiates degradation of connective tissue components like elastin fibers, type IV collagen, proteoglycan, and fibronectin [18]. Furthermore, neutrophil elastase can activate proteinase-activated receptor-2 (PAR2) to trigger acute inflammation and pain within the joints of mice [19]. This response is neurogenic in origin, as activation of PAR2 by neutrophil elastase further results in activation of transient receptor potential vanilloid 4 (TRPV4) to cause sensitization of neurons, inflammation, and pain [20].

Proteinase-activated receptors (PARs) are a specialized group of G-protein coupled receptors (GPCRs) with seven transmembrane loops. Studies have identified four members in the family of PARs which are activated by different serine proteinases via the unveiling of specific tethered ligands [21]. PAR2 is expressed on a variety of inflammatory cells (e.g., mast cells, leukocytes, T and B lymphocytes), and its expression is upregulated when these cells are stimulated, indicating that PAR2 is involved in the pro-inflammatory cascade [22, 23]. PAR2 is also expressed on various joint structures like the synovium, cartilage, chondrocytes, and bone [24–26] which suggests their possible involvement in the structural damage associated with arthritis. Furthermore, PAR2 has been identified on the terminals of joint sensory nerves [27] and their activation leads to the development of joint neurogenic inflammation and pain [27, 28]. Antagonism of PAR2 has been shown to prevent joint inflammation associated with inflammatory arthritis [29]. Furthermore, a deficiency of PAR2 can prevent structural damage associated with surgery-induced OA in mice [30–32].

It is becoming increasingly evident that some pro-inflammatory molecules released into OA joints can initiate nerve damage leading to the generation of joint neuropathic pain [33]; therefore, rapid interventions that can alleviate OA inflammation present an opportunity to abrogate neuronal damage and the subsequent development of neuropathic pain. Intra-articular injection of sodium monoiodoacetate (MIA) inhibits glyceraldehyde-3-phosphate dehydrogenase, which disrupts the glycolytic pathway in chondrocytes and results in cell death [34]. It is a commonly used mouse model for studying OA pain [34]. Several studies have indicated that injection of MIA results in infiltration of inflammatory cells into the synovium leading to a low-grade inflammation which usually resolves within a week [35–39]. These invading neutrophils and macrophages are the likely source of neutrophil elastase in this model [16, 40]. It is feasible, therefore, that this early inflammation may contribute to the pain response that is observed at later time points in this model. Thus, the aim of the present study was to determine whether neutrophil elastase contributes to the development of acute inflammation in the MIA model via activation of PAR2. Secondly, it was hypothesized that inhibition of neutrophil elastase or modulation of PAR2 during the early phase of joint inflammation could alleviate the chronic development of OA pain.

## Methods

### Animals

All experiments were performed on male C57BL/6 mice (Charles River Laboratories Inc., QC, Canada) or wild-type (PAR2+/+) and PAR2-deficient (PAR2−/−) mice raised in-house (original breeders developed on a C57BL/6 background from Jackson Laboratories, Bar Harbor, ME, USA). Following arrival at the animal care facility, mice were allowed to acclimate for at least 1 week. Animal weights ranged from 20 to 34 g (8–14 weeks old). Mice were housed at 22 ± 2 °C on a 12:12 h light/dark cycle with standard lab chow and water available ad libitum. All animal care and experimental procedures complied with the ARRIVE and Canadian Council for Animal Care guidelines (http://www.ccac.ca/) and were approved by the Dalhousie University Committee on Laboratory Animals. A total of 255 animals were used in the experiments described here.

### Assessment of the proteolytic activity of neutrophil elastase

The proteolytic activity of neutrophil elastase was determined using the substrate Neutrophil Elastase 680 FAST (NE 680), a commercially available fluorescence agent that is optically silent upon injection and produces a fluorescence signal after specific clevage by neutrophil elastase [19, 41]. Animals were anesthetized (2–4% isoflurane; 100% oxygen at 1 L/min) and the hair was removed from both hind limbs. NE680 (1 nmol/25 μl) was injected subcutaneously over both knee joints and the mice were immediately placed in a prone position in the imaging chamber of an In-Vivo Xtreme imaging system (Bruker Corporation, Billerica, MA, USA). The substrate was excited at 650 nm wavelength and the emitted fluorescence was captured at 700 nm wavelength for a 2.5-s exposure time; the field of view was 10 cm and the lens aperture (fSTOP) was kept at 2. For analysis, identical regions of interest were drawn around both the ipsilateral (arthritic) and contralateral (naïve) joint, and fluorescence intensities were calculated for each knee. The fluorescence level of the contralateral knee joint was subtracted from that of the inflamed ipsilateral knee joint to account for any release of neutrophil elastase in the skin as a result of the injection itself.

### Intravital microscopy

Intravital microscopy (IVM) was used for the assessment of leukocyte trafficking within the synovial microcirculation, as previously described [19]. Mice were anesthetized using urethane (25% stock solution; 0.2–0.3 ml intraperitoneal (i.p.)) and a surgical plane of anesthesia was confirmed by the absence of a hindpaw withdrawal reflex. Mice were placed in a supine position on a surgery board, and a heated blanket (SoftHeat HP710-24-3P-S Electric Heating Pad, Kaz Inc., Southborough, MA, USA) was used to maintain core body temperature at 37 °C. The trachea was exposed by making a small longitudinal incision in the skin and was cannulated with PE-60 tubing (Clay Adams, Parsippany, NJ, USA) to allow unrestricted breathing. The right carotid artery and jugular vein were then isolated and cannulated with PE-10 tubing (Clay Adams, Parsippany, NJ, USA) filled with heparinized saline (1 U/ml). The carotid artery cannula was connected in series to a pressure transducer (Kent Scientific Corporation, Torrington, CT, USA), and mean arterial pressure was recorded on a differentially amplified blood pressure monitor (BP-1; World Precision Instruments, Sarasota, FL, USA). The fluorescent dye, rhodamine 6G (0.05%; 0.06 ml), was injected through the jugular vein cannula to stain leukocytes immediately before measurement. Lastly, the skin covering the knee joints (∼ 1 cm long × ∼ 0.5 cm wide) was removed and all superficial fasciae removed to allow an unrestricted view of the joint microvasculature. The surface of the knee was perfused intermittently with warm (37 °C) physiological buffer to prevent tissue desiccation. The knee joint microvasculature was visualized under incident fluorescent light using a Leica DM2500 microscope with a HCX APO L 20X objective and a HC Plan 10X eyepiece (Leica Microsystems Inc., Richmond Hill, ON, Canada; final magnification ×200). Straight, unbranched, postcapillary venules (diameter 20–50 μm) were selected for analysis. Recordings of 1 min duration were made using a BC-71 AVT camera (Horn Imaging, Aalen, Germany). Two leukocyte properties were assessed: (i) rolling leukocytes that move along the venular endothelium with a velocity less than the free-flowing cells in the same vessel and (ii) adherent leukocytes within a 100-μm length of venule which cling to the endothelial wall for at least 30 s. Three videos were captured from three different venules per knee joint and the values averaged.

### Behavioral pain assessment

Secondary allodynia was assessed by application of von Frey hair filaments to the plantar surface of the ipsilateral mouse hindpaw using a modification of the Dixon’s up-down method [42]. Animals were placed in elevated Plexiglas chambers on metal mesh flooring and were allowed to acclimate for 20 min. Once the exploratory or grooming behaviour ceased, a von Frey hair filament was applied perpendicular to the plantar surface of the hindpaw (avoiding the toe pads) until the hair started to bend, and the hair was held in place for 3 s. If the mouse showed withdrawal, shaking, or licking of the hindpaw, a positive response was recorded and the next lower strength hair was applied; if there was no response, the next higher strength hair was applied up to a maximum cut-off level which corresponded to a 4-g bending force. After the first difference in response was observed, four more measurements were made and the pattern of responses was converted to a 50% withdrawal threshold calculated using the following formula: 10[*Xf* + *k*δ]/10,000; where *Xf* = value (in log units) of the final von Frey hair used, *k* = tabular value for the pattern of the last six positive/negative responses, and δ = mean difference (in log units) between stimuli.

### Saphenous nerve preparation and G-ratio calculation

Saphenous nerve sections were removed from all pain experiment animals and g-ratios measured. The saphenous nerve preparation and G-ratio calculation were carried out as previously described [33]. Mice were euthanized at the end of the study with sodium pentobarbital (1000 mg/kg, i.p.), and a section of the ipsilateral saphenous nerve above the knee was collected and fixed in 2.5% glutaraldehyde in 0.1 M sodium cacodylate for at least 24 h. After fixation, nerves were rinsed three times using 0.1 M sodium cacodylate buffer. The nerve samples were fixed again with 1% osmium tetroxide for 2 h, washed with distilled water, and kept in 0.25% uranyl acetate at 4 °C overnight. The next day, nerve samples were dehydrated with a graduated series of acetone (50%, 70%, 95%, 100% acetone and lastly by dried 100% acetone beads). Nerve samples were subjected to infiltration with epon araldite resin (3:1 ratio of dried 100% acetone to resin for 3 h, 1:3 ratio of dried 100% acetone to resin overnight, then 100% epon araldite resin 2 times for 3 h). The samples were then embedded in 100% epon araldite resin and placed in 60 °C oven for 48 h to cure. Sections (100 nm thick) were cut using a Reichert–Jung Ultracut E Ultramicrotome with a diamond knife and were placed on copper grids then stained with 2% aqueous uranyl acetate for 10 min followed by two rinses with distilled water for 5 min. These rinsed samples were kept in lead citrate for 4 min and rinsed again with distilled water and left to air dry. The samples were viewed using a JEOL JEM 1230 Transmission Electron Microscope (JEOL, Japan) at 80 kV, and one good quality image was captured per animal using Hamamatsu ORCA-HR digital camera at ×2500 magnification. G-ratio, analyzed using the G-ratio plugin on the ImageJ software (https://imagej.nih.gov/ij), is the square root of the internal axonal area (without myelin) divided by the whole axonal area (including myelin), and is a measure of myelin thickness. All nerve fibers (36 to 113 per section) present in a captured image were measured and were averaged to give a mean g-ratio value for each animal.

### Induction of osteoarthritis

Mice were anesthetized (2–4% isoflurane; 100% oxygen at 1 L/min), and an acceptable plane of anesthesia was confirmed by failure to produce a hindpaw withdrawal reflex. The right knee joint was shaved and baseline knee joint diameter was measured using a digital caliper (Control Company, Friendswood, TX, USA). MIA (0.3 mg/10 μl) was injected into the intra-articular space of the knee joint using a 30-G needle inserted through the patellar ligament. The knee was then manually extended and flexed for 30 s to disperse the MIA throughout the joint. The inflammatory changes (joint diameter and leukocyte trafficking) and behavioral pain were assessed at baseline and on days 1, 3, 7, 10, and 14 post-MIA injection.

### Proteolytic activity of neutrophil elastase in MIA-inflamed knee joints

The proteolytic activity of neutrophil elastase in the inflamed knee joints was determined on days 1 and 14 post-MIA injection. Pharmacologically, the bioactivity of neutrophil elastase can be limited by inhibitors such as sivelestat and serpinA1. Sivelestat (or ONO-5046) is a synthetic, potent, and selective inhibitor of neutrophil elastase with an IC50 of 44 nM [43]. SerpinA1 (or alpha-1 antitrypsin) is a member of the serine proteinase family which primarily inhibits the activity of neutrophil elastase [44] but can also inhibit other serine proteinases including trypsin, chymotrypsin, proteinase 3, and cathepsin G [16]. The dose of sivelestat was selected on the basis of a previous study [19]. SerpinA1 has a long half-life of 15.5 h in mice [45]. The dose of serpinA1 was selected on the basis of pilot experiments in which serpinA1 blocked exogenous neutrophil elastase-induced joint inflammation and pain (data not shown).

A separate cohort of MIA-injected animals was treated on day 1 with the neutrophil elastase inhibitor sivelestat (50 mg/kg i.p.), and proteolytic activity was assessed 4 h later. Another group of MIA-injected animals received treatment with serpinA1 (10 μg i.p.) administered 15 min before and 12 h after MIA injection, and the proteolytic activity was assessed 24 h after injection of MIA.

### Effect of neutrophil elastase inhibition on MIA-induced joint inflammation and pain

To assess the functional contribution of neutrophil elastase in the model of experimental OA, two separate cohorts of animals were injected with MIA. The first cohort received treatment with a synthetic inhibitor of neutrophil elastase, sivelestat (50 mg/kg i.p.), administered 10 min before and 240 min after MIA injection on day 0 and once on days 1 to 3. The second cohort received treatment with an endogenous inhibitor of neutrophil elastase, serpinA1 (10 μg i.p.), administered 15 min before and 12 h after MIA injection. Following treatment, inflammation and pain were assessed at several time points over the 2-week time course.

### Involvement of PAR2 in MIA-induced inflammation and pain

Involvement of PAR2 in MIA-induced inflammation and pain was assessed using the PAR2 antagonist GB83 (5 μg i.p.), administered 10 min before and 120 and 240 min after MIA, on day 0 and once on day 1 (60 min before assessment). MIA was also injected into the knee joints of PAR2 knockout mice. Inflammation experiments focused on day 1, as this is the peak of the MIA-induced inflammatory effect. Behavioral pain was assessed on days 1, 3, 7, 10, and 14 post-MIA injection.

### Statistical analysis

Data are presented as means ± standard error of the mean (SEM) and were analyzed with the statistical software package GraphPad Prism v.6.07 (GraphPad Software Inc., San Diego, CA, USA). The data were first tested for normal distribution using the Kolmogorov–Smirnov test. The inflammation and pain time courses of MIA were analyzed by one-way ANOVA; Dunnett’s post hoc test was used for determining the time point of maximal effect. The inflammation and pain time courses of MIA, with and without sivelestat or serpinA1, were compared using a two-way ANOVA. The fluorescence imaging results were analyzed by one-way ANOVA with Tukey’s multiple comparisons post hoc test. The PAR2 and MIA day 1 inflammation data were analyzed using one-way ANOVA with Dunnett’s post hoc test, comparing all experimental groups to the MIA-treated group. The PAR2 and MIA pain time course data were analyzed by two-way ANOVA. The G-ratio data for the neutrophil elastase inhibitors were analyzed using one-way ANOVA with Dunnett’s post hoc test, comparing all experimental groups to the MIA-treated group. The G-ratio data for PAR2 (+/+) and PAR2 (−/−) mice were analyzed using a Student’s unpaired *t* test.

### Materials

Sodium monoiodoacetate, rhodamine 6G, and urethane were obtained from Sigma-Aldrich (St. Louis, MO, USA). Sivelestat (neutrophil elastase inhibitor; 4-[[[2-[[(carboxymethyl)amino] carbonyl]phenyl]amino]sulfonyl] phenyl ester 2,2-dimethyl-propanoic acid, monosodium salt, tetrahydrate) was obtained from Caymen Chemicals (Ann Arbor, MI, USA). SerpinA1 (neutrophil elastase inhibitor) was obtained from Abcam, Inc. (Toronto, ON, Canada). GB83 (PAR2 antagonist; N-((S)-3-cyclohexyl- 1-((2S,3S)-1-(2,3-dihydrospiro[indene-1,4′-piperidine]-1′-yl)- 3-methyl-1-oxopentan-2-ylamino)-1-oxopropan-2-yl) isoxazole-5-carboxamide) was obtained from Axon Medchem (Groningen, The Netherlands). Neutrophil Elastase 680 FAST was purchased from PerkinElmer (Waltham, MA, USA). Sodium monoiodoacetate, sivelestat, and rhodamine 6G were dissolved in saline. GB83 was dissolved in vehicle (1:1:8 DMSO/cremophor/saline). Physiological buffer (composition—135 mM NaCl, 20 mM NaHCO3, 5 mM KCl, 1 mM MgSO4*7H2O, pH = 7.4) was prepared in-house.

## Results

### Proteolytic activity of neutrophil elastase in MIA-induced inflamed knee joints

**Fig. 1:**
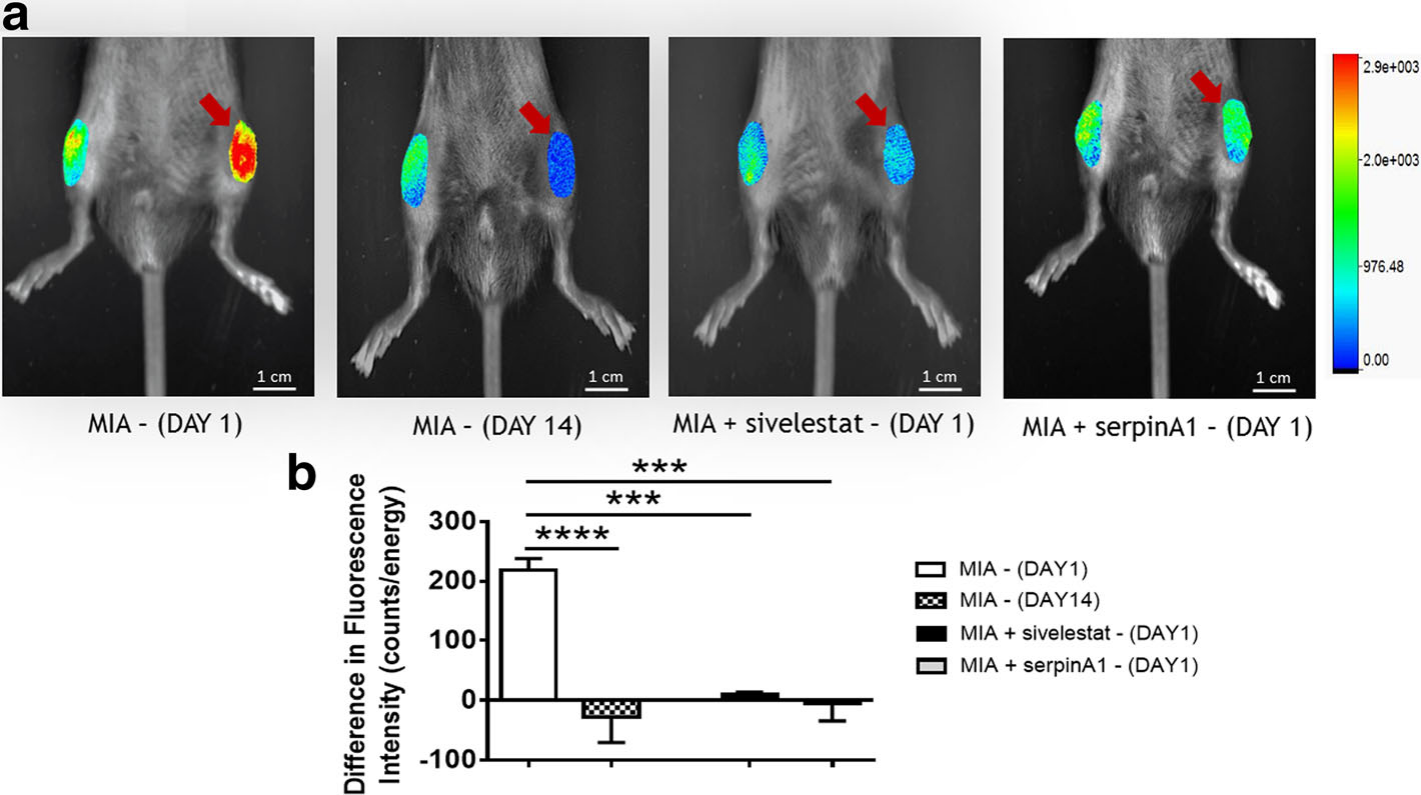
In vivo imaging of neutrophil elastase enzyme activity in MIA-injected knee joints and the effect of neutrophil elastase inhibition with sivelestat (50 mg/kg i.p., 20 h post-injection of MIA, i.e., 4 h before imaging) or serpinA1 (10 μg i.p., administered 15 min before and 12 h after MIA injection, i.e., 12 h before imaging). **a** Representative fluorescence images showing the area of interest analyzed for MIA-injected knees (*red arrow*) vs. contralateral naïve knee joints. **b** Intra-articular injection of MIA produced an increase in proteolytic activity on day 1 that was not present by day 14; treatment with sivelestat or serpinA1 caused a significant decrease in day 1 neutrophil elastase activity (*n* = 5–7 per group). ***P < 0.001, ****P < 0.0001 compared to the MIA control, one-way ANOVA

The proteolytic activity of neutrophil elastase within the knee joint was increased on day 1 after MIA injection, and this effect was significantly reduced by treatment with either sivelestat or serpinA1 (Fig. 1b, *P* < 0.001, one-way ANOVA with Tukey’s test, *N* = 5–7). By day 14, the proteolytic activity of neutrophil elastase was low compared to day 1 (Fig. 1b, *P* < 0.0001).

### Effect of neutrophil elastase inhibition on MIA-induced inflammation and pain

**Fig. 2:**
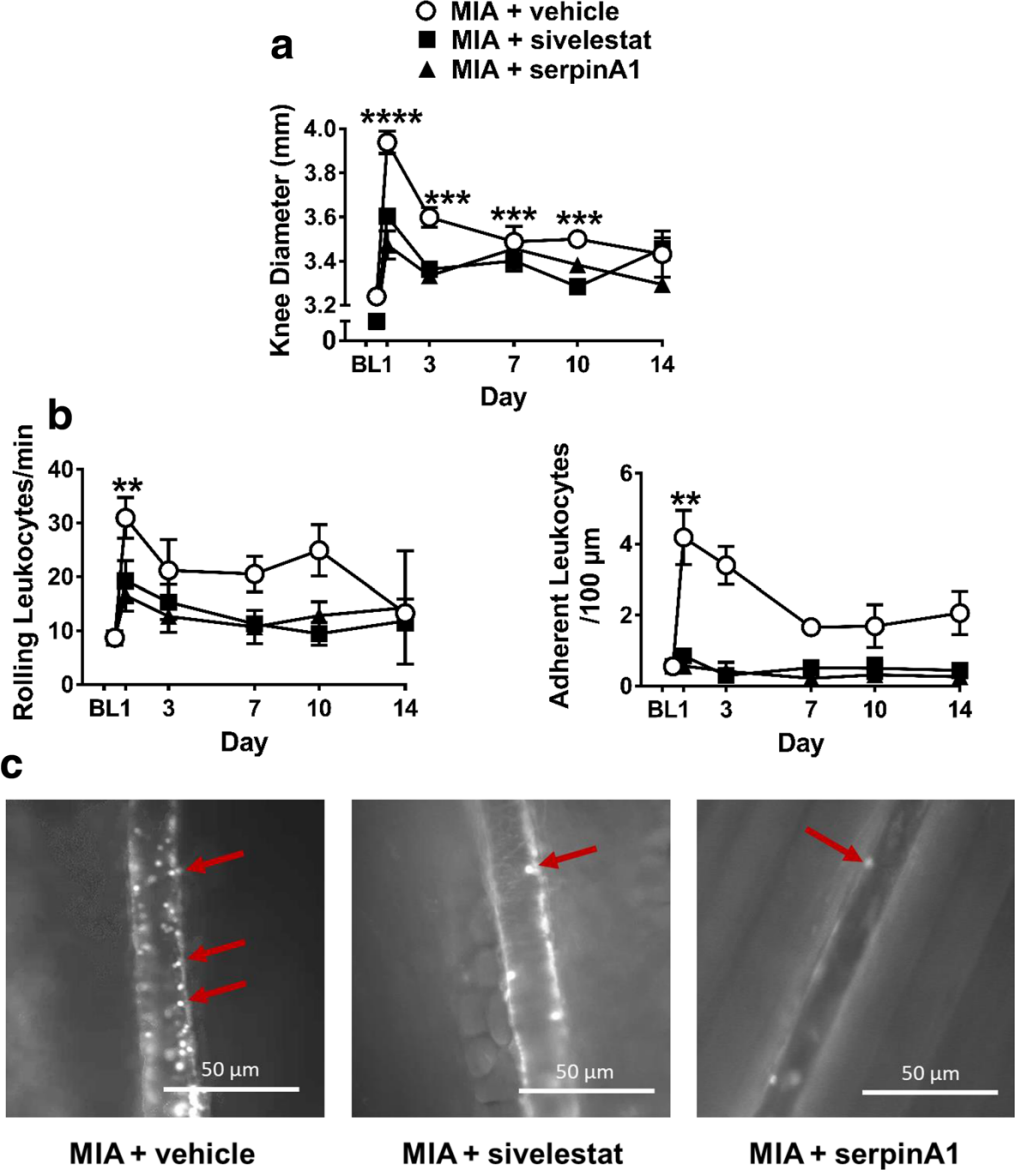
Time course of inflammation induced by intra-articular injection of MIA (0.3 mg/10 μl on day 0) and the effect of neutrophil elastase inhibition with sivelestat (50 mg/kg i.p., administered 10 min before and 240 min after MIA injection on day 0 and once on days 1 to 3) or serpinA1 (10 μg i.p., administered 15 min before and 12 h after MIA injection). **a** MIA induced a significant increase in knee joint diameter at 1, 3, 7 and 10 days post-injection that was blocked by systemic treatment with sivelestat (*P* < 0.0001, two-way ANOVA) or serpinA1 (*P* < 0.0001, two-way ANOVA). The number of **b** rolling and adherent leukocytes were increased 1 day after intra-articular injection of MIA, and these increases were inhibited by sivelestat (*P* < 0.01, *P* < 0.001, respectively, two-way ANOVA) and serpinA1 (*P* < 0.05, *P* < 0.001, respectively, two-way ANOVA) (*n* = 5–28 per time point). **P < 0.01, ***P < 0.001, and ****P < 0.0001, as compared to baseline, one-way ANOVA. *Lower panel* **c** shows representative intravital micrographs illustrating the inhibitory effect of neutrophil elastase blockade on synovial leukocyte trafficking; *red arrows* indicate stained leukocytes

Compared to baseline, intra-articular injection of MIA produced a significant increase in knee joint diameter on days 1, 3, 7, and 10 (Fig. 2a, *P* < 0.001, one-way ANOVA, *N* = 6–28). The number of rolling (Fig. 2b, *P* < 0.01, *N* = 24) and adherent (Fig. 2b, *P* < 0.01, *N* = 27) leukocytes increased significantly on day 1 as compared to baseline then decreased over the remainder of the 2-week time course. Treatment with sivelestat (on days 0–3) blocked the increase in knee diameter (Fig. 2a, *P* < 0.0001, two-way ANOVA, *N* = 5–28 per time point), number of rolling leukocytes (Fig. 2b, *P* < 0.01), and number of adherent leukocytes (Fig. 2b, *P* < 0.001) across the time course. Likewise, treatment with serpinA1 (on day 0) also blocked joint edema (Fig. 2a, *P* < 0.0001), number of rolling leukocytes (Fig. 2b, *P* < 0.05), and number of adherent leukocytes (Fig. 2b, *P* < 0.001) across the time course. Example images showing the inhibitory effect of neutrophil elastase blockade on leukocyte trafficking are shown in Fig. 2c.

**Fig. 3:**
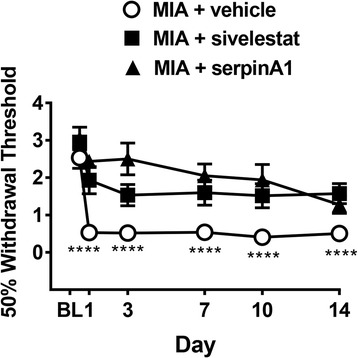
Time course of joint mechanosensitivity induced by intra-articular (i.artic.) injection of MIA (0.3 mg/10 μl on day 0) and the effect of neutrophil elastase inhibition with sivelestat (50 mg/kg i.p., administered 10 min before and 240 min after MIA injection on day 0 and once on days 1 to 3) or serpinA1 (10 μg i.p., administered 15 min before and 12 h after MIA injection). MIA injection produced a significant decrease in tolerance to tactile stimulation throughout the 14-day time course which was inhibited by early treatment with sivelestat (*P* < 0.0001, two-way ANOVA) and serpinA1 (*P* < 0.0001, two-way ANOVA) (*n* = 8–9 per time point). ****P < 0.0001 as compared with baseline, one-way ANOVA

Intra-articular injection of MIA caused a significant decrease in hindpaw mechanosensitivity, indicative of secondary allodynia. This pain appeared on day 1 and persisted to day 14 post-injection (Fig. 3, *P* < 0.0001, one-way ANOVA, *N* = 8–9 per time point). Moreover, early blockade of neutrophil elastase using sivelestat or serpinA1 decreased MIA-induced allodynia throughout the time course (Fig. 3, *P* < 0.0001, two-way ANOVA, *N* = 8–9 per time point).

### Involvement of PAR2 in mediating MIA-induced inflammation and pain

**Fig. 4:**
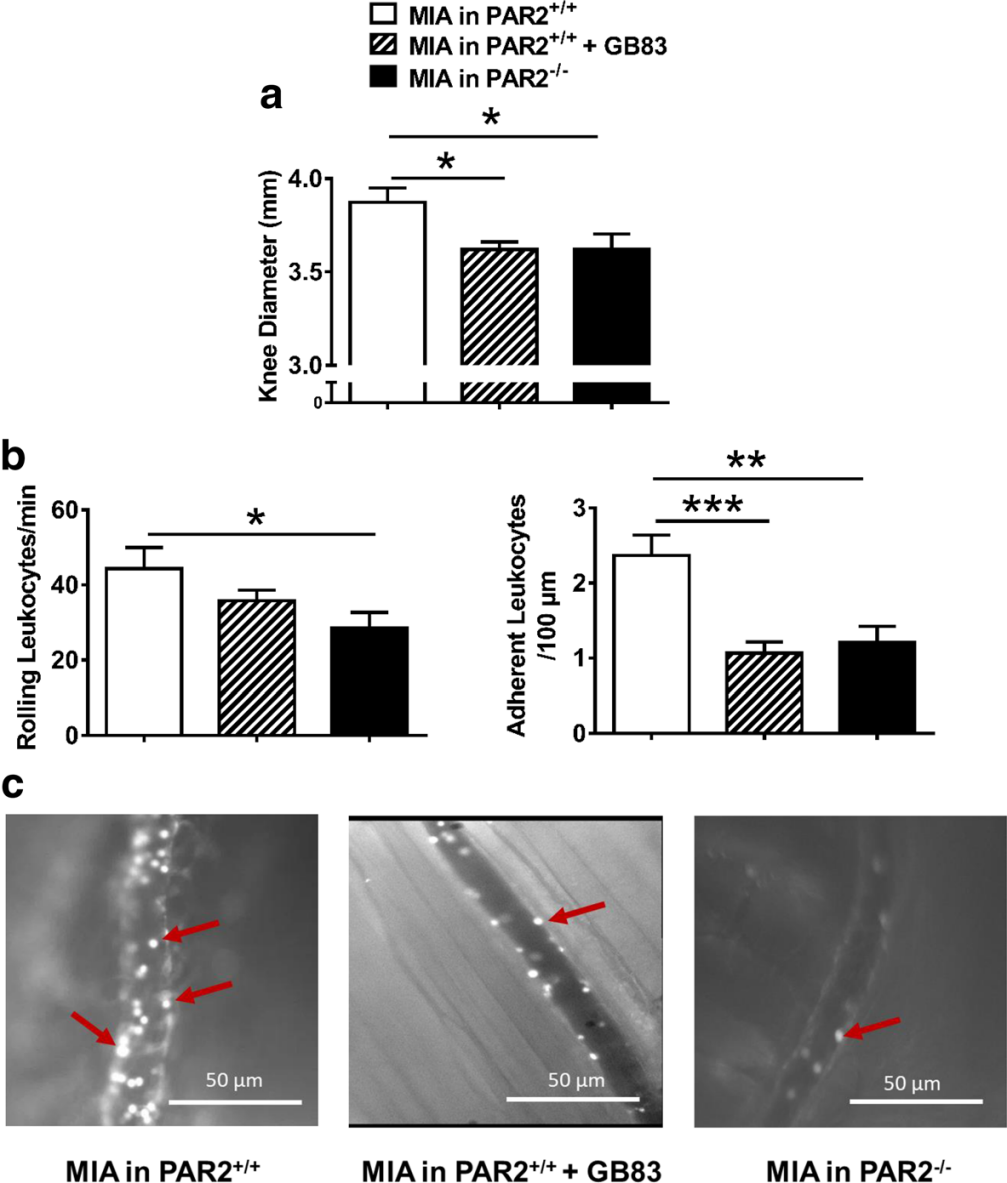
Involvement of PAR2 in mediating MIA-induced joint inflammation. **a** The MIA-induced increase in knee joint diameter on day 1 post-injection was blocked by systemic treatment with GB83 (5 μg i.p., administered 10 min before and 120 and 240 min after MIA, on day 0 and once on day 1 (60 min before assessment) and absent in PAR2 knockout (PAR2^−/−^) mice. **b** The increased number of rolling leukocytes were reduced in PAR2 knockout mice. **b** The increased number of adherent leukocytes on day 1 was reduced by treatment with GB83 (5 μg i.p., administered 10 min before and 120 and 240 min after MIA, on day 0 and once on day 1 (60 min before assessment)) and absent in PAR2 knockout mice. (*n* = 8–9 per group) *P < 0.05, **P < 0.01, ***P < 0.001, compared to the MIA control, one-way ANOVA. **c** *Lower panel* shows representative intravital micrographs in the different cohorts of mice; *red arrows* indicate stained leukocytes

The PAR2 antagonist GB83 (administered on days 0 and 1) significantly blocked the day 1 MIA-induced increase in knee joint diameter (Fig. 4a, *P* < 0.05, one-way ANOVA with Dunnett’s post hoc test; *N* = 9), and the number of adherent leukocytes (Fig. 4b, *P* < 0.001). In PAR2 knockout mice, MIA failed to cause a day 1 increase in knee joint diameter (Fig. 4a, *P* < 0.05), number of rolling leukocytes (Fig. 4b, *P* < 0.05), or number of adherent leukocytes (Fig. 4b, *P* < 0.01, *N* = 9). Intravital images showing the involvement of PAR2 in leukocyte trafficking during this acute phase of MIA are shown in Fig. 4c.

**Fig. 5:**
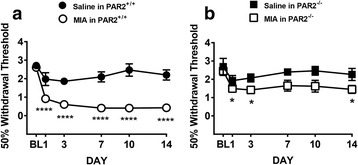
Time course of joint mechanosensitivity induced by intra-articular injection of MIA (0.3 mg/10 μl) or saline (10 μl) and involvement of PAR2. **a** In wild-type mice, MIA induced a significant reduction in tolerance to tactile stimuli that was not seen with an intra-articular saline injection (*P* < 0.0001, two-way ANOVA). **b** MIA induced mechanical sensitivity in PAR2 knockout (PAR2^−/−^) mice (*P* < 0.001, two-way ANOVA) that was less intense than in wild-type mice. **a**, **b** There were no differences in response to saline injection between the wild-type and PAR2 knockout mice (*P* = 0.61, two-way ANOVA); however, a significant difference was observed between wild-type and PAR2 knockout mice injected with MIA (*P* < 0.0001, two-way ANOVA) (*n* = 5–10 per group). *P < 0.05, ****P < 0.0001 as compared with baseline, one-way ANOVA

In pain assessment experiments, intra-articular injection of MIA caused significant hindpaw allodynia which appeared on day 1 and persisted to day 14 post-injection in wild-type mice (Fig. 5a, *P* < 0.0001, *N* = 5–10). Intra-articular injection of saline had no effect on hindpaw mechanosensitivity over the 2-week time course in wild-type (Fig. 5a) or PAR2 knockout (Fig. 5b) mice. In PAR2 knockout animals, MIA caused a mild secondary allodynia on days 1, 3, and 14 (Fig. 5b, *P* < 0.05, *N* = 10). However, the secondary allodynia observed in PAR2 knockouts was significantly less severe as compared to wild-type mice (Fig. 5, *P* < 0.0001), suggesting knockout of PAR2 partially prevents the development of pain caused by MIA.

### Effect of neutrophil elastase inhibition on MIA-induced saphenous nerve demyelination

**Fig. 6:**
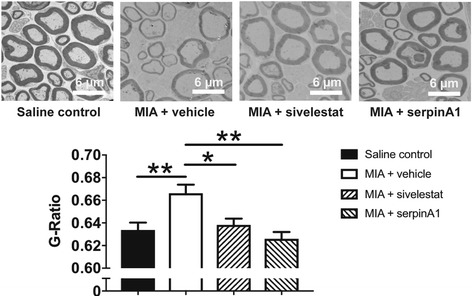
Involvement of neutrophil elastase in MIA-induced saphenous nerve demyelination. *Upper panels* show representative electron micrographs and *lower panel* shows assessment of myelin thickness of neurons from mouse saphenous nerves 14 days after intra-articular injection of MIA or saline. Injection of MIA (0.3 mg/10 μl day 0) caused significant demyelination (increased G-ratio) compared to injection of saline (10 μl day 0). Systemic treatment with the neutrophil elastase inhibitors sivelestat (50 mg/kg i.p., administered 10 min before and 240 min after MIA injection on day 0 and once on days 1 to 3) or serpinA1 (10 μg i.p., administered 15 min before and 12 h after MIA injection) prevented demyelination. (*n* = 5–8 per group) *P < 0.05, **P < 0.01, compared to the MIA control, one-way ANOVA

Injection of MIA resulted in a significant increase in G-ratio values as compared to saline-injected controls (Fig. 6, *P* < 0.01, one-way ANOVA with Dunnett’s post hoc test; *N* = 8), indicating demyelination of the saphenous nerve fibers. Early blockade of neutrophil elastase using sivelestat (Fig. 6, *P* < 0.05) or serpinA1 (Fig. 6, *P* < 0.01) blocked MIA-induced demyelination.

**Fig. 7:**
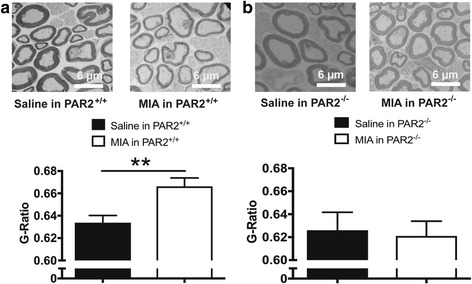
Involvement of PAR2 in MIA-induced saphenous nerve demyelination. *Upper panels* show representative electron micrographs and *lower panels* show assessment of myelin thickness of neurons from mouse saphenous nerves 14 days after intra-articular injection of MIA or saline. Separate cohorts of wild-type (**a**) and PAR2 knockout (**b**) mice were injected with MIA (0.3 mg/10 μl) or saline (10 μl) on day 0. MIA caused significant demyelination (increased G-ratio) of the saphenous nerves of wild-type mice (**a**) but not of PAR2 knockout mice (**b**) (*n* = 5–10 per group), **P < 0.01, Student’s unpaired *t* test

Compared to saline-injected wild-type mice, MIA resulted in significant demyelination of the saphenous nerve fibers, as evidenced by an increase in G-ratio (Fig. 7a, *P* < 0.01, unpaired *t* test, *N* = 8). No such demyelination was observed in PAR2 knockout mice (Fig. 7b, *P* = 0.81, *N* = 5–10).

## Discussion

The results presented here demonstrate a transient inflammatory response in the early stages of the MIA model of OA which is, in part, mediated by neutrophil elastase. Imaging studies clearly show that neutrophil elastase is proteolytically active in MIA-treated knee joints on day 1 which abates by day 14. Systemic treatment with the neutrophil elastase inhibitor sivelestat or serpinA1 significantly reduced the proteolytic activity of neutrophil elastase on day 1 post-MIA injection, confirming that these drugs can inhibit the MIA-induced increase in neutrophil elastase.

Synovitis is known to occur in a subgroup of OA patients which is short-lasting, intermittent, and associated with episodes of intense pain. In this study, injection of MIA caused acute pro-inflammatory changes within the knee joint, as evidenced by an increase in knee joint diameter and leukocyte trafficking. The inflammation peaked on day 1, decreased by day 3, and remained at a low level for the remainder of the study period. These findings are consistent with previous studies where MIA produced significant edema and pain when compared to intra-articular saline [35, 36]. Guzman et al. [46] showed that the edematous fluid procured from day 1 MIA joints contained fibrin, protein, and infiltrated inflammatory cells; this inflammatory exudate subsided by day 7 after MIA injection. Various cytokines (TNF-α, IL-1β, IL-6) and adhesion molecules (ICAM-1, VCAM-1, P-selectin) are involved in the extravasation of leukocytes at the site of inflammation [47]. These leukocytes release neutrophil elastase which can cleave important adhesion molecules and activate pro-inflammatory cytokines, thereby contributing to further leukocyte adhesion and extravasation [48–51].

Sivelestat and serpinA1 can directly inhibit the enzymatic activity of neutrophil elastase, and can reduce inflammation [52–58]. In the present study, sivelestat and serpinA1 inhibited the activity of neutrophil elastase during the early, acute inflammatory phase of the MIA model. These agents decreased joint edema and reduced the number of rolling and adherent leukocytes following treatment, suggesting that neutrophil elastase is present and contributes to leukocyte extravasation in the early inflammatory phase of MIA-induced OA.

Injection of MIA leads to an immediate and lasting pain response in ipsilateral hindlimbs. Acute, early treatment with sivelestat or serpinA1 not only blocked this mechanical hypersensitivity during the treatment phase but prevented development of a chronic pain response at later time points. These results suggest that early inhibition of neutrophil elastase to block acute inflammation can inhibit the later development of pain in this model of OA. Additionally, a recent investigation by Vicuna et al. [59] confirmed that neutrophil elastase, secreted by infiltrating T cells within the dorsal root ganglia, contributes to the development of neuropathic pain after nerve injury. There is substantial evidence to show that MIA has the capacity to cause nerve injury, as indicated by an upregulation of the nerve injury marker activating transcription factor 3 (ATF-3) [38, 60–62]. The results described here show that intra-articular MIA causes demyelination of saphenous nerve fibers and suggest that the nerve damage and mechanical pain that develop in the MIA model of OA is, at least in part, related to the early presence of neutrophil elastase. It is possible that the neutrophil elastase inhibitors could also attenuate joint damage associated with OA; however, this was not tested here as the MIA model does not fully recapitulate the degenerative characteristics seen in human OA.

Several studies have indicated that neutrophil elastase evokes inflammation and pain via activation of PAR2 [19, 20, 63]. Activation of PAR2 can trigger inflammation in various tissues like the gut, airways, and joints [23, 63–65]. Indeed, activation of PAR2 leads to several inflammatory changes such as increased cytokine production, joint edema, leukocyte-endothelial interactions, upregulation of intercellular adhesion molecules, and synovial hyperemia [19, 27, 28, 65, 66]. In the experiments described here, the PAR2 antagonist GB83 inhibited edema and leukocyte trafficking in MIA-injected knee joints. This pharmacological observation was corroborated in PAR2 knockout mice. Neutrophil elastase-induced inflammation has previously been shown to be blocked by GB83 and absent in PAR2 knockout mice [19]. It follows, therefore, that the local release of endogenous neutrophil elastase likely leads to activation of articular PAR2 to induce knee joint inflammation in MIA-injected mice.

MIA-induced secondary allodynia was reduced in the absence of PAR2 suggesting this receptor plays an important role in the development of pain in this model. It is known that PAR2, SP, and CGRP are co-expressed on dorsal root ganglion neurons [67, 68], suggesting an interplay between PARs and inflammatory neuropeptides. Since MIA can cause the peripheral release of SP and CGRP [12], it is feasible that the pain associated with PAR2 activation is related to neurogenic inflammation. The data presented here show a reduction in MIA-induced secondary allodynia after inhibition of neutrophil elastase. It is, therefore, likely that endogenous neutrophil elastase is causing proteolytic cleavage of PAR2 to elicit secondary allodynia in MIA-induced arthritis, as neutrophil elastase-induced pain has also been shown to be PAR2-dependent [19].

G-ratio analysis revealed that injection of MIA reduced myelin thickness in the saphenous nerve fibers innervating the knee joints of wild-type mice. Intriguingly, there was no loss of myelin in PAR2 knockout mice, which suggests that this receptor is involved in joint peripheral neuropathy.

## Conclusions

The data presented here show that neutrophil elastase is bioactive in the early phase of the mouse MIA model. This enzyme contributes to the initial inflammatory response associated with this model and promotes the later development of joint pain and neuropathy. Pharmacological and knockout experiments suggest that these effects are mediated by cleavage of PAR2. The exact mechanisms responsible for the development of inflammation, pain, and nerve damage after PAR2 activation by neutrophil elastase and the role of other PAR2 activating serine proteinases requires further investigation. These data highlight the potential utility of early treatment with neutrophil elastase inhibitors and/or PAR2 blockers to reduce the development of OA pain at later stages of the disease.

## Abbreviations

ATF-3: Activating transcription factor 3
CGRP: Calcitonin gene-related peptide
i.p.: Intraperitoneal
IC50: Half maximal inhibitory concentration
ICAM-1: Intercellular adhesion molecule
IL-1β: Interleukin 1 beta
IL-6: Interleukin 6
IVM: Intravital microscopy
MIA: Sodium monoiodoacetate
NE 680: Neutrophil elastase 680 FAST
OA: Osteoarthritis
PAR2: Proteinase-activated receptor-2
SEM: Standard error of the mean
SP: Substance P
TNF-α: Tumor necrosis factor alpha
TRPV4: Transient receptor potential vanilloid 4
VCAM-1: Vascular cell adhesion protein 1
